# In-depth characterization of protein N-glycosylation for a COVID-19 variant-design vaccine spike protein

**DOI:** 10.1007/s00216-023-04533-w

**Published:** 2023-01-26

**Authors:** Jiangming Huang, Shouzeng Hou, Jiao An, Chenliang Zhou

**Affiliations:** Shanghai Zerun Biotech Co., Ltd, Shanghai, China

**Keywords:** COVID-19 spike protein, Variant, Glycosylation, Mass spectrometry

## Abstract

COVID-19 is caused by SARS-CoV-2 infection and remains one of the biggest pandemics around the world since 2019. Vaccination has proved to be an effective way of preventing SARS-CoV-2 infection and alleviating the hospitalization burden. Among different forms of COVID-19 vaccine design, the spike protein of SARS-CoV-2 virus is widely used as a candidate vaccine antigen. As a surface protein on the virus envelop, the spike was reported to be heavily N-glycosylated and glycosylation had a great impact on its immunogenicity and efficacy. Besides, N-glycosylation might vary greatly on different expression systems and sequence variant designs. Therefore, comprehensive analysis of spike N-glycosylation is of great significance for better vaccine understanding and quality control. In this study, full characterization of N-glycosylation was performed for a Chinese Hamster Ovary (CHO) cell expressed variant-designed spike protein. The spike protein featured the latest six-proline substitution design together with the incorporation of a combination of mutation sites. Trypsin and Glu-C digestion coupled with PNGase F strategies were adopted, and effective LC–MS/MS methods were applied to analyze samples. As a result, a total of 19 N-glycosites were identified in the recombinant pike protein at intact N-glycopeptide level. Quantitative analysis of released glycan by LC–MS/MS was also performed, and 31 high-abundance N-glycans were identified. Sequencing analysis of glycan was further provided to assist glycan structure confirmation. Moreover, all of the analyses were performed on three consecutive manufactured batches and the glycosylation results on both glycosite and glycans showed good batch-to-batch consistency. Thus, the reported analytical strategy and N-glycosylation information may well facilitate studies on SARS-CoV-2 spike protein analysis and quality studies.

**Graphical Abstract:**

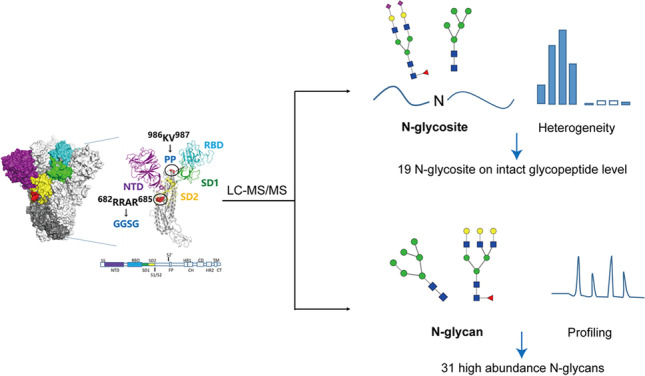

**Supplementary Information:**

The online version contains supplementary material available at 10.1007/s00216-023-04533-w.

## Introduction

Since the emergence of novel coronavirus disease (COVID-19, caused by severe acute respiratory syndrome coronavirus 2, SARS-CoV-2) in December 2019, it has caused vast infections and deaths worldwide. According to the latest WHO report, there have been 551,226,298 confirmed cases of COVID-19, including 6,345,595 deaths (by 5:33 p.m. CEST, 8 July 2022, https://covid19.who.int/) and the number is still increasing, causing great loss to human health and the economy globally. Meanwhile, COVID-19 vaccination has been proved to be an effective way to prevent virus infection and alleviate hospitalization burden. Among different commercially available and late-clinical-stage COVID-19 vaccines, it can be found that the viral envelop protein, the Spike, was most commonly used as a candidate vaccine antigen [1]. According to structural biology studies, the Spike protein was presented on the surface of SARS-CoV-2 in a trimeric form [2]. It was involved in the process of interaction with angiotensin-converting enzyme 2 (ACE-2) upon viral invasion. Therefore, structural characteristics such as epitopes, mutations, and glycosylation were expected to play important roles in the protein functionality and may affect the quality of COVID-19 vaccines [3].

Several studies have been carried out to explore the biological function of protein glycosylation of Spike in different variants of concern (VOC) and variants of interest (VOI). For example, Zhang et al., 2022, found that loss of the N370 glycosylation by T372A mutation increased about eightfold binding affinity to ACE2 and thus enhanced SARS-CoV-2 infectivity [4]. Ye et al., 2021, reported that N- and O-glycosylation on the receptor binding domain (RBD) of the Spike protein showed significant impact on antibody binding. It may lead to an incomplete neutralization effect and impact the immunogenic integrity of RBD‐based (one domain of the Spike) vaccines [5]. Beaudoin et al., 2022, found that the unique N679K mutation in the Omicron strain increased the propensity for O-linked glycosylation at the S1/S2 cleavage site and prevent recognition by proteases. Such glycosylation in the Omicron strain may hinder entry at the cell surface and decrease syncytia formation, inducing cell entry through the endocytic pathway [6]. Therefore, glycosylation of the Spike protein may have great impact on both the antigenicity and immunogenicity of the COVID-19 vaccine.

Despite being an important protein post-translational modification, glycosylation is highly diverse and remains difficult to be analyzed [7]. The most common types of glycosylation are N-glycosylation and O-glycosylation. N-Glycosylation occurs on the first asparagine residue with specific amino acid sequence motif of N-X-S/T (X can be any amino acid except proline), and bears a core penta-oligosaccharide of Man3GlcNAc2 structure; O-glycosylation normally refers to complex glycan attached to serine or threonine residues [8]. To date, high-performance liquid chromatography coupled with tandem mass spectrometry (HPLC–MS/MS) remained a powerful technique for protein glycosylation analysis. Liquid chromatography provides superior separation performance for complicated glycopeptide or glycan mixtures generated by enzymatic digestion, while tandem mass spectrometry offers accurate measurement of molecular weight. Furthermore, tandem mass spectrometry provides molecular fragment information and may assist structure or composition analysis [9–12]. Thus, high-throughput and high-resolution analysis of glycosite, glycan, or even intact glycopeptide can be achieved.

Much effort has been made to unravel the complex glycosylation pattern of the SARS-CoV-2 Spike protein. For example, Watanabe et al., 2020, firstly reported systematic analysis of glycosylation for an early SARS-CoV-2 Spike protein (GenBank: MN908947) expressed in the 293F cell line, and provided detailed glycosylation information mapping to the trimeric structure [13]. Besides, Spike protein expressed in diverse cell lines such as 293, Calu-3, or Baculovirus insect cells has also been studied and a plethora of mass spectrometry techniques such as EthcD have been tested to enhance glycosylation analytical performance [14–16]. However, since the Spike protein has a large molecular weight with 22 potential N-glycosites, diverse mutation sites, and different vaccine sequence designs and expression systems, analysis of its glycosylation remains tedious and difficult [17]; therefore, the development of a more robust analytical strategy and the generation of more information on different variant designs and expressed Spike protein remain largely in demand.

In this study, we performed detailed glycosylation analysis to a Spike protein that has a special sequence design for a broadly protective COVID-19 vaccine. The candidate Spike protein was expressed in the CHO-K1 cell line and purified to a relatively high purity (≥ 95%, assessed by size-exclusion HPLC). Different protease and peptide N-glycosidase F (PNGase F)–based sample preparation strategies were surveyed and LC–MS/MS analytical methods optimized to enhance Spike protein glycosylation analytical performance. Site-specific glycan, intact glycopeptide, and overall N-glycan profiling were analyzed to provide comprehensive identification of Spike protein glycosylation. Finally, the optimized analytical method was applied to three consecutive manufactured batches to evaluate analytical performance and to unravel more glycosylation information of the distinct Spike as a candidate vaccine protein.

## Materials and methods

### Materials

The recombinant variant-design Spike protein of SARS-CoV-2 was expressed in the CHO-K1 cell line. The CHO-K1 cell line selected for this product came from European Collection of Authenticated Cell Cultures (ECACC) (No. 85051005). Zhongshan Kangtian Shenghe Biotechnology Co., Ltd., QuaCell, one of our partners, obtained the original adherent CHO-K1 cell from Public Health England (PHE) in England. The cells were domesticated in serum-free and suspension culture (protected by patent application) by them, and the cell lines have also been used in previous publications [18, 19]. Glu-C and trypsin, both sequencing grade, were purchased from Roche and Sigma-Aldrich, respectively. Other reagents, such as peptide-N-glycosidase F (PNGase F, New England Biolabs), iodiacetamide (IAM, Sigma-Aldrich), guanidine hydrochloride (Gu.HCl, Invitrogen), and DL-dithiothreitol (DTT, VWR) were all from commercial sources.

### Methods

#### Glycopeptide preparation

The Spike protein (400 μg) was dissolved into Gu.HCl (8 M) to a final concentration of 0.5 mg/mL and was incubated with DTT (25 mM) at 37 ℃ for 30 min with stirring. The sample was alkylated with IAM (50 mM) for 30 min in the dark at room temperature. The resulting mixture was ultra-filtered to transfer the protein into a phosphate-buffered saline solution (PBS, 25 mM, pH 7.59) with a total volume of 400 μL. The solution was aliquoted into four, two of which were digested directly (with Glu-C or trypsin, 25:1 w/w, 37 ℃ for 21 h with stirring), and the other two were treated with PNGase F (25:1 w/w, 37 ℃ for 22 h with stirring) before digestion (with Glu-C or trypsin, 25:1 w/w, 37 ℃ for 21 h with stirring). The proteolytic reactions were quenched by adding formic acid to a final volume ratio of 1%. The mixtures were centrifuged at 12,000 rpm for 5 min, and the supernatants were transferred to new vials for LC–MS analysis.

#### LC–MS analysis of glycopeptide

Reverse-phase chromatography coupled to electrospray ionization tandem mass spectrometry (LC–MS/MS) was performed on an Orbitrap Q Exactive mass spectrometer connected to a Vanquish™ Flex UHPLC (Thermo Scientific). The protein digest was separated on a C18 column (ACQUITY UPLC Peptide BEH, 300 Å, 1.7 μm, 2.1 mm × 150 mm). The mobile phase was 0.1% formic acid in ddH_2_O (double-distilled water, mobile phase A) and 0.1% formic acid in acetonitrile (mobile phase B) with a 90-min gradient with mobile phase B ranging 1 to 90%. The flow rate was 0.3 mL/min, and 20 μL of the digests was injected. Online desalting was achieved with a divert valve that switched LC from waste to MS detection at 1.5 min.

Mass spectrometry (MS) data acquisition was performed using a high-energy collision dissociation (HCD) method. MS1 scans were performed at a resolution of 70,000 over m/z 200–2000, and the automatic gain control (AGC) was set as 3e6, with the maximum injection time of 200 ms. Data-dependent HCD tandem mass spectra were acquired at a resolution of 17,500 with normalized collision energies (NCE) of 20, 30, and 40%.

#### Analysis of the N-glycan profile

To release N-glycan, PNGase F was added to a PBS solution of the Spike protein (1:25, w/w, incubated at 37 ℃ for 21 h with stirring), which was denatured, reduced, and alkylated through the same treatment as glycopeptide preparation. The deglycosylated protein was precipitated with pre-cold ethanol. After centrifugation at 12,000 rpm for 5 min, the supernatant was transferred to a new vial and the solvent was removed in a centrifugal vacuum concentrator. The N-glycans were re-dissolved and labeled with 2-AB labeling solution (50 μL of 0.37 M 2-aminobenzamide and 0.95 M sodium cyanoborohydride in 30% v/v acetic acid in dimethyl-sulfoxide) at 65 ℃ for 2 h. After being concentrated to dryness in a centrifugal vacuum concentrator, the residue was dissolved in 30 μL ddH_2_O and 70 μL acetonitrile stepwise. The mixture was centrifuged at 12,000 rpm for 5 min, and the supernatant was transferred to a new vial for LC–MS analysis.

#### LC–MS analysis of the N-glycan profile

Hydrophilic interaction chromatography (HILIC) coupled to electrospray ionization tandem mass spectrometry (LC–MS/MS) was performed on an Orbitrap Q Exactive mass spectrometer connected to a Vanquish™ Flex UHPLC (Thermo Scientific). The labeled glycans were separated on an ACQUITY BEH Amide column (130 Å, 1.7 μm, 2.1 mm × 150 mm). The mobile phase was 50 mM ammonium formate in ddH_2_O (pH 4.4, mobile phase A) and acetonitrile (mobile phase B) with the following gradient: 75–54% B (0–51.9 min); 0% B (53.4–56 min); 0–75% B (56–59.9 min); 75% B (63.8–71.6 min); and 0% B (76.8 min). The flow rate was 0.4 mL/min except for a change to a lower rate of 0.2 mL/min in the time interval from 53.4 to 59.9 min, and 20 μL of the labeled glycans was injected.

Mass spectrometry data acquisition was performed using a high-energy collision dissociation (HCD) method. MS1 scans were performed at a resolution of 70,000 over m/z 380–4000, and the automatic gain control (AGC) was set as 3e6, with the maximum injection time of 200 ms. Data-dependent HCD tandem mass spectra were acquired with a resolution of 17,500 with normalized collision energies (NCE) of 20%, 30%, and 40%.

#### Data analysis for glycopeptide identification and N-glycan profiling

The MS data were analyzed against the known protein sequence for confirmation and post-translational modifications (PTMs) using the Thermo BioPharma Finder 3.2 and pGlyco 3.0 software, with variable modifications: Deamidated (N), Deamidated (Q), Oxidation (MW), Phosphorylation (STY), Glycation (K), and N/O-glycan (CHO), and fixed modifications: Carbamidomethyl (C) and pyro-Glutamic (N-termQ). For N-glycan profiling analysis, raw data were analyzed by Xcalibur software (version 4.1.31.9) to obtain monoisotopic masses. Glycan structure was generated with the free glycoworkbench software [20]. Glycopeptide mass spectrometry data and search results were deposited on the public database iProx (www.iprox.org), an official member of ProteomeXchange Consortium [21–23]. The Subproject iProx ID was IPX0005157001 and the PXD is PXD038220.

## Results and discussions

**Fig. 1:**
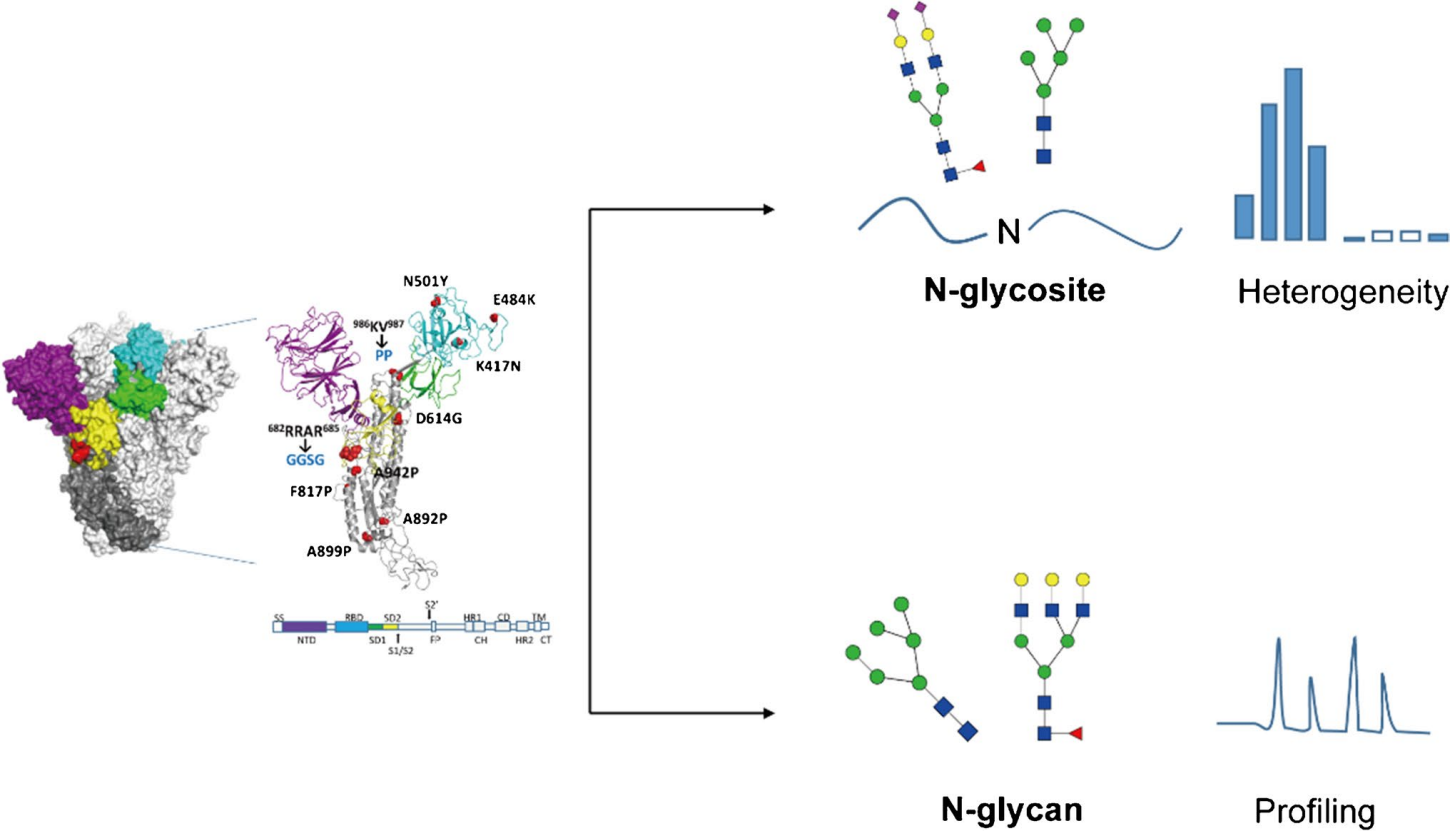
Depiction of the overall analytical strategy of Spike protein N-glycosylation

The Spike protein to be analyzed in this study was specially designed with the six-proline substitution (F817P, A892P, A899P, A942P, K986P, and V987P) [24], furin cleavage site replacement (^682^RRAR^685^ to GGSG linker), and a combination of variant mutation sites such as K417N, E484K, N501Y, and D614G, as compared to the wild-type COVID-19 Spike protein sequence. The original signal peptide of Spike protein (MFVFLVLLPLVSS) was also replaced with a strong one (MEFGLSWLFLVAILKGVQC) for recombinant production in CHO-K1 cells. The specific design of Spike had a sequence length of 1228 amino acids, with a trimeric foldon in its C-terminal to enhance trimerization. Besides, according to our internal study, no inter-chain disulfide bond was formed (data not shown). The Spike protein contains a total of 22 potential N-glycosites based on its theoretical sequence. Therefore, with a relatively high molecular weight and multiple glycosylation sites, full analysis of N-glycosylation of the specific design of Spike protein remains challenging. In this work, a generic LC–MS/MS-based pipeline was developed to comprehensively characterize the Spike protein N-glycosylation, as is shown in Fig. 1. For protein N-glycosite analysis, intact glycopeptides were well identified and the relative percentage of glycan on each site was calculated by Biopharma Finder™ 3.2 Software (Thermo Scientific). For each site, normalized percentages of each site-specific glycan were reported for both glycosylated (%intact GP) and de-N-glycosylated (%deN GP) sample. Detailed calculations of the percentage were provided in the S-1 part of Electronic supplementary material 1. It should also be noted that current intact glycosylation identification still relied heavily on data interpretation tools. For N-glycan profiling analysis, glycan was released from the Spike protein and labeled with 2-AB to enhance MS ionization efficiency and the qualitative and quantitative analyses were mainly achieved by vendors’ software and total ion chromatogram integration.

Overall, the presented work was more of adopting traditional methods with some optimization for better analysis of the distinct Spike protein sample. Optimized analytical methods were applied on three homemade continuous-manufactured lots to evaluate analytical performance and batch-to-batch consistency.

### Analysis of N-glycosite for three parallel batches

For N-glycosylated site analysis, the strategy of different protease digestion was firstly optimized to tackle the sequence coverage problem. Due to the complexity of the SARS-CoV-2 Spike protein sequence, some of the potential N-glycosylated sites such as N590, N603, N644, N696, N704, et al. are prone to be missing when only trypsin is used due to long digested peptide or multiple glycosites on a single peptide. The case was deteriorating even further for N-glycosylation analysis when six-proline substitution was introduced into the Spike protein. Therefore, different proteases and digestion procedures were tested and a dual protease with glycosidase analytical strategy was finally adopted. In short, aliquoted protein was digested firstly by trypsin and Glu-C, respectively, to generate different peptide mixtures. One half of the digested peptides were further de-N-glycosylated (termed “deN” in this work) while the other half was left untreated (termed “intact”). High-resolution LC–MS/MS analysis was performed to each sample, and MS data were generated. The Biopharma Finder™ 3.2 software (Thermo Fisher) is capable of processing two-conditioned LC–MS data and can also report glycosylation proportion on each site.

As a result, nearly 100% of sequence coverage was obtained for all three tested lots by the dual protease strategy that ensured full coverage of all potential N-glycosites, as shown in Table 1. The strategy was successfully applied to three lots of purified Spike protein drug substance. The base peak chromatograms of LC–MS/MS, as depicted in Fig. S1-6 in Electronic supplementary material 1, also showed good digestion performance and batch-to-batch consistency.

**Table 1:** Overall MS identification performance of three parallel lots

Lot no	Protease	With deglycosylation	Sequence coverage	Missed N-sites, single	Total sequence coverage (%)	Missed N-sites, total
Lot 1	Trypsin	No	92.35%	6	99.43%	0
		Yes	98.13%	2		
	GluC	No	81.45%	3		
		Yes	84.30%	1		
Lot 2	Trypsin	No	92.35%	6	99.43%	0
		Yes	98.13%	1		
	GluC	No	81.94%	3		
		Yes	84.62%	1		
Lot 3	Trypsin	No	92.35%	6	100.0%	0
		Yes	97.72%	2		
	GluC	No	82.75%	3		
		Yes	88.12%	1		

With the dedicated design of the experiment, a total of 19 N-glycosylated sites were successfully identified at the intact N-glycopeptide level, namely N4, N109, N136, N152, N221, N269, N318, N330, N590, N603, N644, N696, N704, N788, N1061, N1085, N1121, N1145, and N1181, as shown in Table S1 in Electronic supplementary material 2. All identified sites were also highly confirmed with current Uniprot entry of P0DTC2 (https://www.uniprot.org/uniprotkb/P0DTC2/entry). For each glycosylated site, varied glycans were identified and provided comprehensive information of abundant site-specific glycan. Similar site-specific glycan patterns were found as compared roughly to that of Spike expressed in the HEK293F cell line by Watanabe et al., 2020 [13]. For example, N4, N136, N318, N644, and N1145 were dominated by a complex-type glycan; N221 was mainly highly mannosylated, and N109, N788, and N1061 showed a mixture of three glycan types; however, on our CHO-expressed variant-design Spike protein, N152, N269, N330, and N1085 contained a higher degree of high-mannose-type glycan. The glycosylation differences might come from the expression system or distinct protein sequence design. It should be noted that the N-site sequence number in this work was 13 smaller than Watanabe’s because signal peptide was not included.

For the remaining 3 N-glycosites, N48, N61, and N1160, no intact glycopeptide was reported by Biopharma Finder. Manual inspection of extracted ion chromatogram changes before and after PNGase F treatment for site N48/N61 and N1160 were performed. As a result, the extracted ion chromatography of the deamidated peptide SSVLHSTQDLFLPFFS**N**_48_VTWFHAIHVSGT**N**_61_GTK (5 + , m/z 736.5674) increased greatly from 6.40E4 to 1.84E7 (Fig. S7 in Electronic supplementary material 1), indicating both sites were N-glycosylated. While for N1160, peptide sequence **N**_1160_ISGINASVVNIQKE (2 +) in either unglycosylated form (736.4096 ± 10 ppm) or deamidated form (736.9023 ± 10 ppm) showed similar order of abundance magnitudes and the TIC abundance of the unglycosylated form was much higher than that of the deamidated form (Fig. S8-9 in Electronic supplementary material 1). These evidences indicated that site N1160 was unglycosylated or with low site occupancy.

**Fig. 2:**
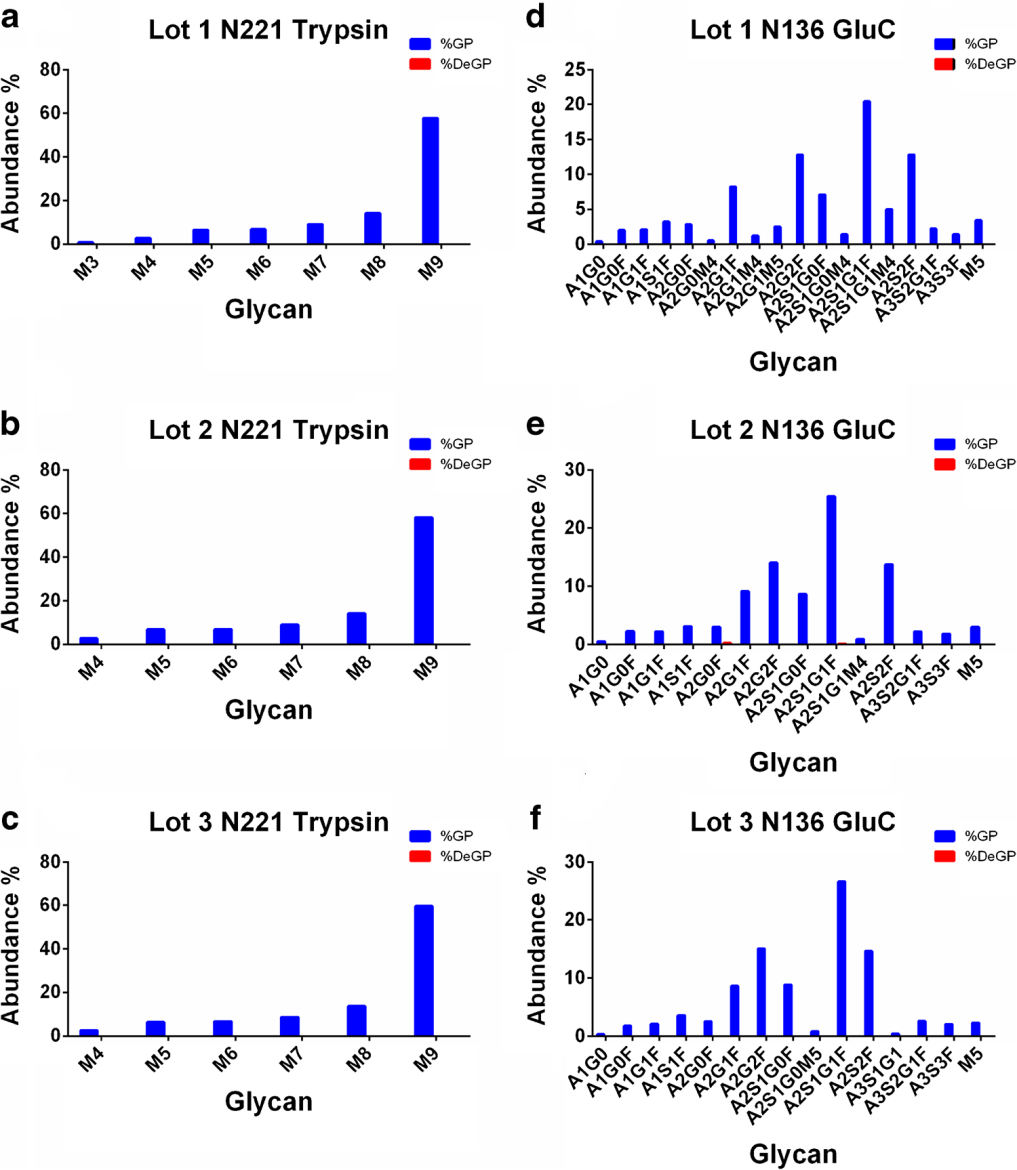
Demonstration of the different site-specific glycopeptide identification performances of trypsin and gluc digestion followed by PNGase F treatment (red bars) or not (blue bars) for N221 (**a**–**c**) and N136 (**d**–**f**)

Figure 2 further demonstrated distinct advantages of utilizing different proteases in N-glycosylation site identification. For N-glycosite N221, tryptic digestion generated several site-specific glycans on the sequence of DLPQGFSALEPLVDLPIGI**N**_221_ITR, while no intact N-glycopeptide bear site N221 was reported under GluC digestion. High-mannose type-N glycans (M3–M9) were found on this site; for N-glycosite N136, however, tryptic digestion samples reported no N-glycopeptides, while dozens of complex-type N-glycan were successfully identified only on the peptide FQFCNDPFLGVYYHKN**N**_136_KSWME generated by GluC treatment.

Cases might be that poor glycopeptide characterization performance may be recovered by different proteolytic peptides. This analytical strategy might also help in the analysis of the Spike protein and other similar glycoproteins. It was worthy of mention that the relative glycan abundance on each site highly resembled across different lots; for example, on site N109 identified by tryptic digestion, a series of complex-type and high-mannose-type N-glycans were reported with the highest glycan being M5 (Man5GlcNAc2) across three tested lots, thus showing good batch-to-batch consistency even on the site-specific glycopeptide level.

**Fig. 3:**
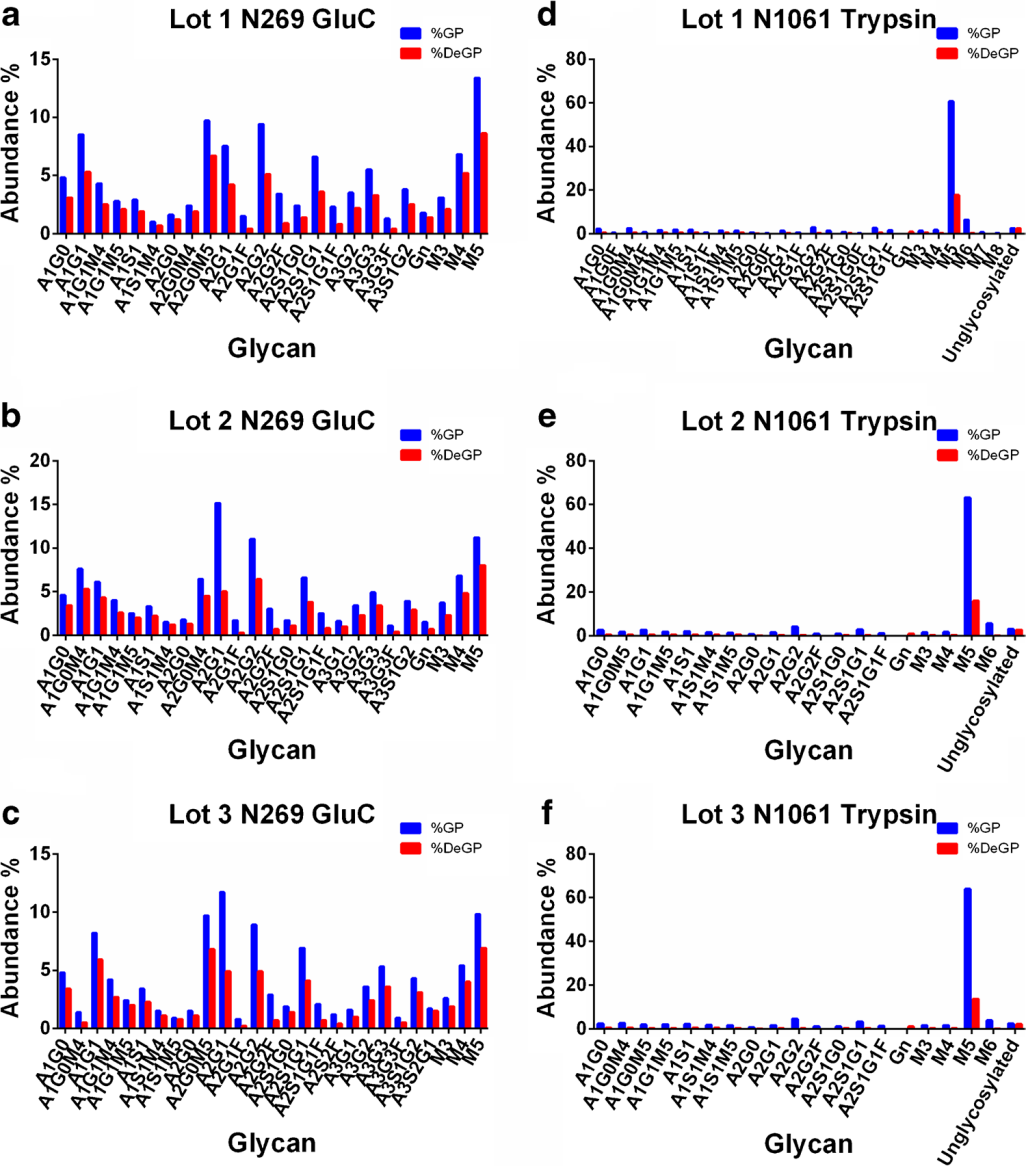
Residual glycan observed on two glycosites by gluc and trypsin digestion followed by PNGase F treatment (red bars) or not (blue bars) for N269 (**a**–**c**) and N1061 (**d**–**f**)

Although both N-glycosylation and O-glycosylation have been reported to have happened on the Spike, O-glycosylation remains highly challenging due to highly diverse site locations and different lengths of O-glycan, ranging from one GalNAc to large glycan moieties. In this work, pGlyco3 [25] was also applied to analyze the protein O-glycosylation for each deglycosylated data. As a result, 3 potential O-glycosites (T271, T1063, and T1064) were all reported across 3 batches. A detailed identification list was provided in Table S2 in Electronic supplementary material 2. As shown in Fig. 3, both N269 and N1061 showed a large quantity of uncleaved glycan with either trypsin or GluC digestion condition respectively. For identified glycopeptide bearing N269 by GluC digestion, the peptide sequences were “**N**_269_GTITD” and its missed cleavage form of “**N**_269_GTITDAVDCALDPLSE”; for N1061 by tryptic digestion, the identified peptide sequence was “**N**_1061_FTTAPAICHDGK.” With careful inspection, a common glycosylated Asn was found on the N-terminus of each peptide. It was reported previously that peptides with glycosylated Asn at the N-terminus showed resistance to PNGase F treatment [26, 27]. Therefore, residual glycosylation on both sites may also be ascribed to N-terminal N-glycosylation generated by distinct protease digestion. Further inspection showed that corresponding glycopeptide forms (YNE**N**_269_GTITDAVDCALDPLSETK by trypsin and K**N**_1061_FTTAPAICHD, K**N**_1061_FTTAPAICHDGKAHFPRE by Glu-C) were fully deglycosylated as shown in Table S1 in Electronic supplementary material 2, indicating the de-N-glycosylation on these glycopeptides was effective. These results also exhibited that our complementary protease strategy helped in the in-depth identification of Spike N-glycosylation. Besides, as full removal of N-glycosylation was a prerequisite of pGlyco3, the remaining glycan on N269 and N1061 might well interfere with the data search of pGlyco3. Taking into consideration these results, no O-glycosylation could be confidently reported by our study. Given that Spike O-glycosylation identification is still challenging due to low abundance and lack of consensus O-glycan or conserve sequence motif [28], further identification of the Spike O-glycosylation was still under development and was not discussed in this work.

### Analysis of N-glycan profiling for three parallel batches

**Fig. 4:**
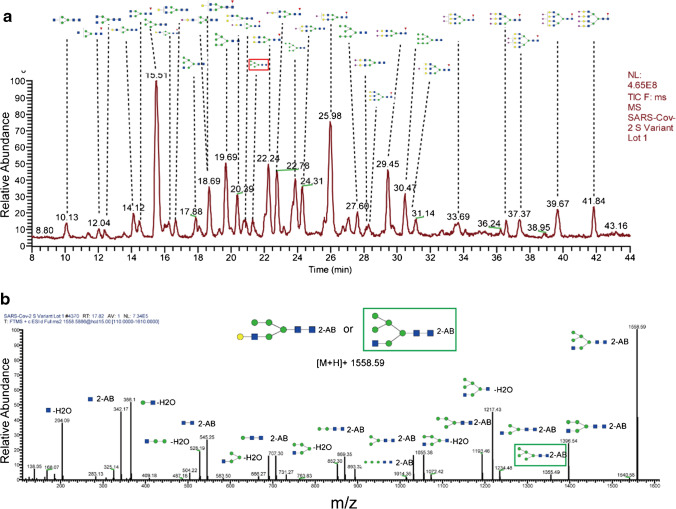
**a** Total ion chromatogram–based N-glycan profiling of the lot 1 sample and depiction of identified glycan of each peak with the percent peak area higher than 0.5%. **b** Example of a tandem mass spectrum for glycan with an m/z of 1558.59 (glycans were all plotted by the Glycoworkbench software)

N-Glycan profiling was generally accepted as an important quality attribute of recombinant protein drugs or vaccines. While an intact glycopeptide level was not easy to be accurately quantified due to micro-heterogeneity of peptide backbones, glycan profiling has been well used for quantitation and batch-to-batch analysis. In this work, an HILIC-LC–MS method was performed to identify N-glycan profiling of the variant design of Spike protein. As a result, a total of 31 N-glycans were confidently identified by the HILIC-LC–MS (Fig. 4a, Table S3 in Electronic supplementary material 2) for peaks with a percentage peak area higher than 0.50% within 8.00 to 44.00 min. The 2-AB labeling efficiency was relatively high with only one identified glycan (percentage peak area of 1.63%) that failed to be derived. Detailed analysis of the lot 1 sample N-glycan profiling showed that about 22.73% (by intensity-based percentage peak area) identified N-glycan contained core-fucosylated and about 30.15% glycan contained sialic acid. Analysis of glycan type further showed that most of the glycan was of complex type (74.36%) with antennary ranging from 2 to 4, and high-mannose type (M5–M9, 22.51%), while only a trace proportion (1.50%) was ascribed to hybrid-type N-glycan (Fig. S10 in Electronic supplementary material 1). The identified glycan was also confirmed with aforementioned site-specific analysis such as M5 et al. Besides, analysis of three consecutive batches showed that purified drug substance showed good quantitative performance and superior lot-to-lot consistency of N-glycan profiling (Fig. S11 in Electronic supplementary material 1), thus greatly ensuring good drug quality. Further demonstration of the variability of percent glycan area for the three biological replicate analysis was provided in Fig. S12 in Electronic supplementary material 1.

Since oligosaccharides are highly isomerized, identification merely based on total molecular weight in most cases cannot fully differentiate structures with different glycans with the same molecular weight. What is worse, as a newly emerged antigen protein and vaccine candidate, there is still a lack of international variant-design Spike protein standard, not to mention distinct N-glycan standard for the Spike. Here, we showed the superior qualitative ability of tandem mass spectrometry in further elucidating glycan substructures as an effective and economic supplement. Based on an optimized high-energy collision parameter of LC–MS/MS, good fragmentation of glycan was obtained. Figure 4b demonstrated well the identification of the glycan Hex5HexNAc2. Two glycan isomers had the same m/z of 1558.59. However, according to the MS2 spectrum, the presence of 1355.49 (outlined in green) can only be generated by terminal HexNAc, thus excluding the first assumptive structure (HexNAc non-terminated). Moreover, the consecutive fragmentation patterns could also assist well the confirmation of glycan substructure and provide more information of glycan. All reported glycan was identified with good tandem mass spectra for each lot.

## Conclusions

In this work, a set of LC–MS/MS-based analytical strategies were performed and dedicatedly optimized to fully unravel protein N-glycosylation patterns of a variant-design Spike protein. Dense N-glycosylation was found on the Spike expressed in the CHO-K1 cell line. The reported site-specific glycan analysis and N-glycan profiling demonstrated protein N-glycosylation from two aspects, that is, the micro-heterogeneity and total glycan abundance. The two aspects could make good complementation to each other in confirming protein N-glycosylation. Since recombinant Spike protein remains a promising and effective vaccine for prevention of COVID-19, better understanding of its glycosylation is of vital importance for vaccine manufacture and quality control. The analytical methods such as multiple protease digestion, intact N-glycopeptide comparison, and tandem mass spectrometry–based glycan characterization reported in this work were expected to be universal and should shed light on better analysis of N-glycosylation for other important glycoproteins of interest.

## Supplementary Information

Below is the link to the electronic supplementary material.

Supplementary file1 (DOCX 2985 KB)

Supplementary file2 (XLSX 163 KB)
