# Supplementary material for: In-depth characterization of protein N-glycosylation for a COVID-19 variant-design vaccine spike protein
Source: Anal Bioanal Chem. 2023 Jan 26;415(8):1455–64. doi: 10.1007/s00216-023-04533-w (PMC9878482; doi:10.1007/s00216-023-04533-w)
Supplement: Supplementary file 1 — Supplementary file1 (DOCX 2985 KB) [file 216_2023_4533_MOESM1_ESM.docx]

**Analytical and Bioanalytical Chemistry**

**Electronic Supplementary Materials-1**

**In Depth Characterization of Protein Glycosylation for a COVID-19 Variant-Design Vaccine Spike Protein**

Jiangming Huang1#, Shouzeng Hou1#, Jiao An1, Chenliang Zhou1*

1. Shanghai Zerun Biotech Co., Ltd., Shanghai, China.

# These authors contributed equally to this work

* Chenliang Zhou, zhouchenliang@walvax.com

**Content**

**S-1** Explanation of the % Abundance column and its calculation

**Fig. S1** Base peak chromatogram (BPC) of lot 1 sample using Trypsin followed by PNGase F treatment (b) or not (a), red shadings indicate peptide identification.

**Fig. S2** Base peak chromatogram (BPC) of lot 1 sample using GluC followed by PNGase F treatment (b) or not (a), red shadings indicate peptide identification.

**Fig. S3** Base peak chromatogram (BPC) of lot 2 sample using Trypsin followed by PNGase F treatment (b) or not (a), red shadings indicate peptide identification.

**Fig. S4** Base peak chromatogram (BPC) of lot 2 sample using GluC followed by PNGase F treatment (b) or not (a), red shadings indicate peptide identification.

**Fig. S5** Base peak chromatogram (BPC) of lot 3 sample using Trypsin followed by PNGase F treatment (b) or not (a), red shadings indicate peptide identification.

**Fig. S6** Base peak chromatogram (BPC) of lot 3 sample using GluC followed by PNGase F treatment (b) or not (a), red shadings indicate peptide identification.

**Fig. S7** (a) Total ion chromatogram (TIC) for Lot 1 sample with tryptic digestion; (b) TIC for Lot 1 sample with PNGase F and tryptic digestion; (c) Extracted ion chromatogram (XIC) of m/z 736.5674 ± 10 ppm for Lot 1 sample with tryptic digestion; (d) XIC of m/z 736.5674 ± 10 ppm for Lot 1 sample with PNGase F and tryptic digestion.

**Fig. S8** (a) Total ion chromatogram (TIC) for Lot 1 sample with GluC digestion; (b) TIC for Lot 1 sample with PNGase F and GluC digestion; (c) Extracted ion chromatogram (XIC) of m/z 736.4096 ± 10 ppm for Lot 1 sample with GluC digestion; (d) XIC of m/z 736.4096 ± 10 ppm for Lot 1 sample with PNGase F and GluC digestion.

**Fig. S9** (a) Total ion chromatogram (TIC) for Lot 1 sample with GluC digestion; (b) TIC for Lot 1 sample with PNGase F and GluC digestion; (c) Extracted ion chromatogram (XIC) of m/z 736.9023 ± 10 ppm for Lot 1 sample with GluC digestion; (d) XIC of m/z 736.9023 ± 10 ppm for Lot 1 sample with PNGase F and GluC digestion.

**Fig. S10** Pie chart and percentage of different N-glycan types of lot 1 N-glycan profiling data

**Fig. S11** Total ion chromatograms of lot 1, lot 2 and lot 3 samples (from top to bottom) N-glycan profiling

**Fig. S12** Depiction of N-glycan percent area and variation for 3 tested Lots.

**S-1** Explanation of the % Abundance column and its calculation

According to the vendor’s instruction of *Chapter 15 Viewing the Modification Summary Page* in the *Thermo BioPharma Finder User Guide* (Software Version 3.2, XCALI-98114), the % Abundance columns (one for each raw data file used in the experiment) display the abundance of the modification in the sample as a percentage.

For a non-targeted peptide mapping experiment, the application uses the following equation

when calculating the values in this column for a particular raw data file:

$$\% Abundace=\frac{Sum of the MS area for all modifed components}{Sum of the MS area for all seleted components}\times100$$

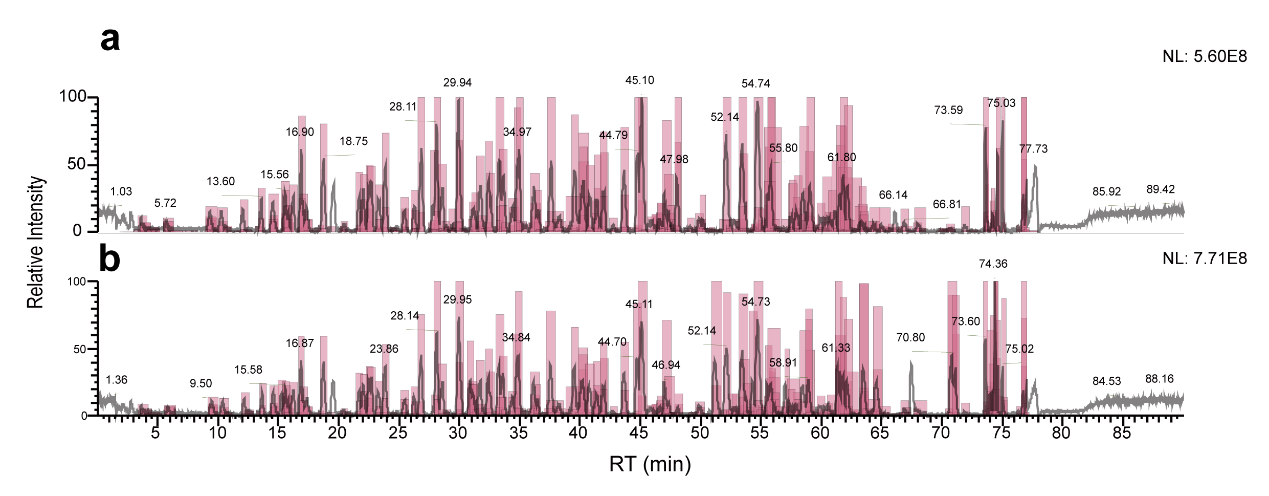


**Fig. S1** Base peak chromatogram (BPC) of lot 1 sample using Trypsin followed by PNGase F treatment (b) or not (a), red shadings indicate peptide identification.


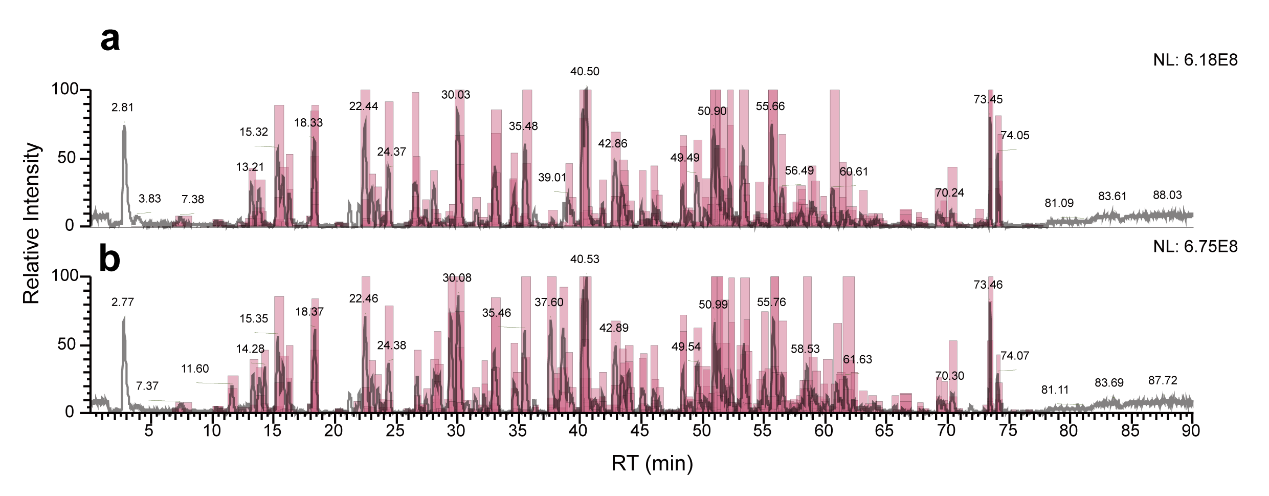


**Fig. S2** Base peak chromatogram (BPC) of lot 1 sample using GluC followed by PNGase F treatment (b) or not (a), red shadings indicate peptide identification.


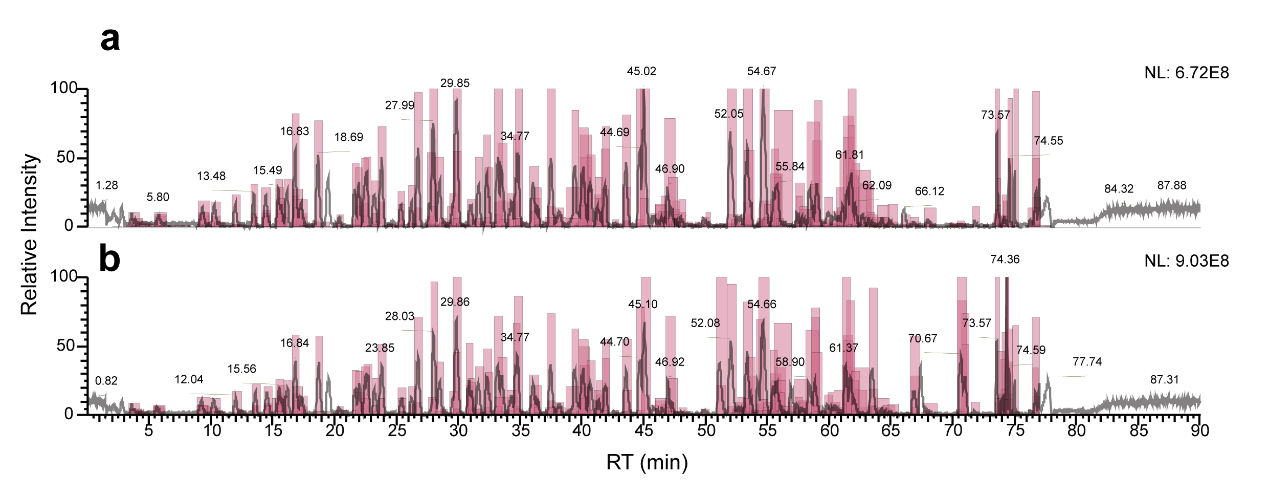


**Fig. S3** Base peak chromatogram (BPC) of lot 2 sample using Trypsin followed by PNGase F treatment (b) or not (a), red shadings indicate peptide identification.


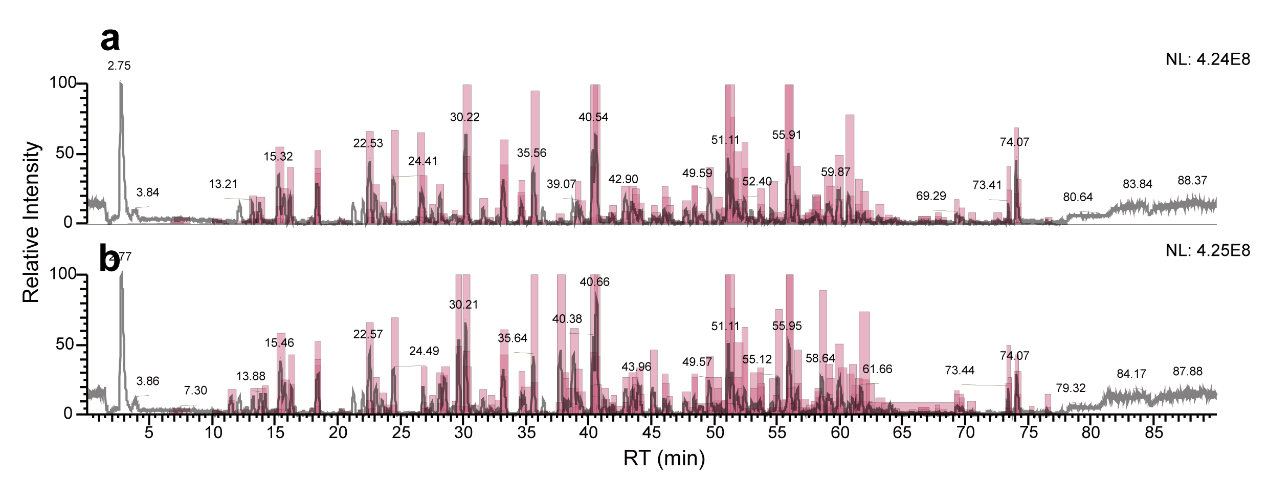


**Fig. S4** Base peak chromatogram (BPC) of lot 2 sample using GluC followed by PNGase F treatment (b) or not (a), red shadings indicate peptide identification.


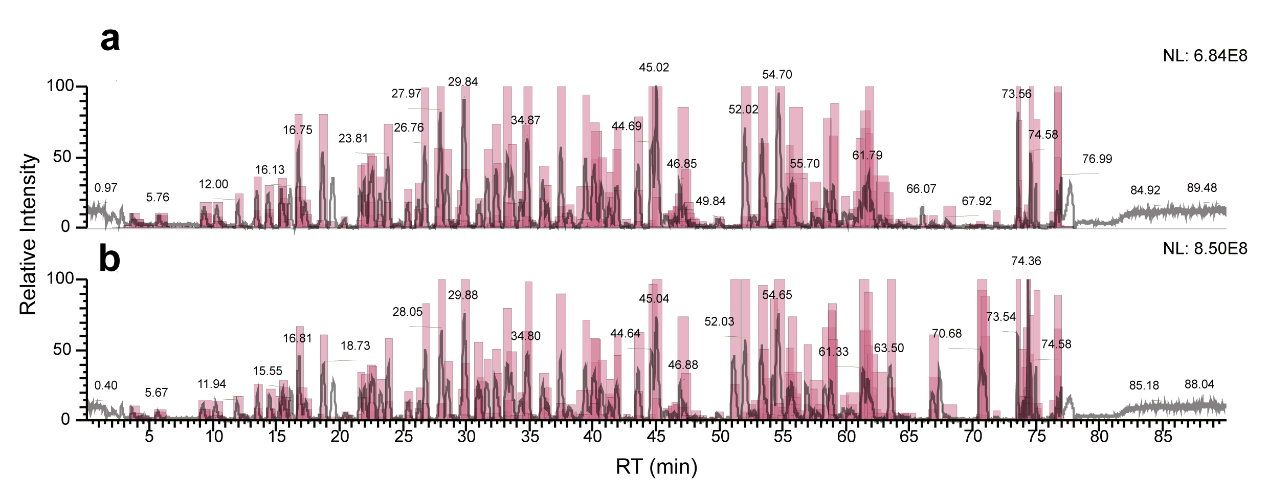


**Fig. S5** Base peak chromatogram (BPC) of lot 3 sample using Trypsin followed by PNGase F treatment (b) or not (a), red shadings indicate peptide identification.


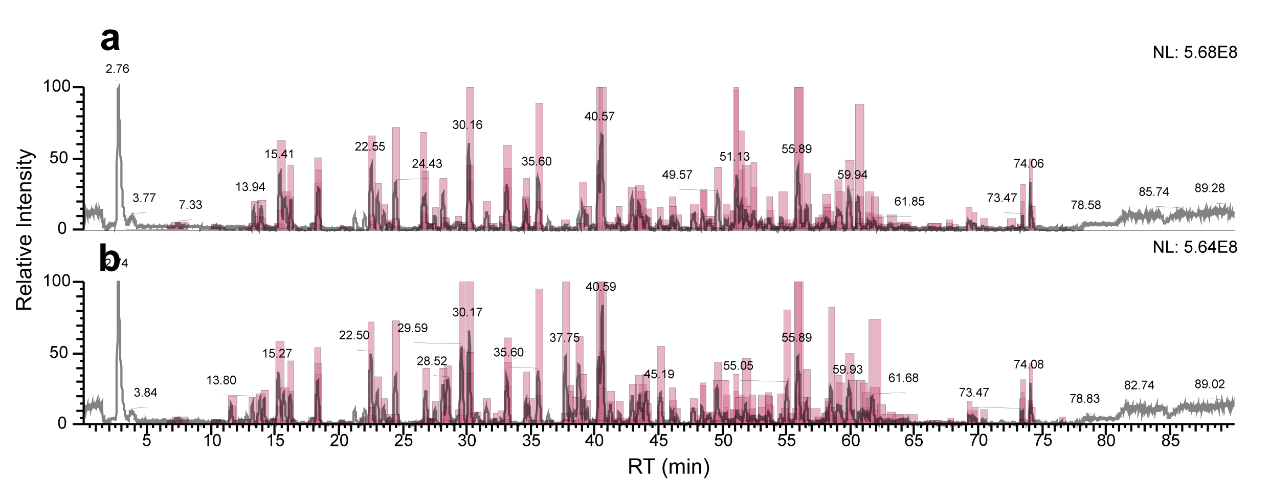


**Fig. S6** Base peak chromatogram (BPC) of lot 3 sample using GluC followed by PNGase F treatment (b) or not (a), red shadings indicate peptide identification.


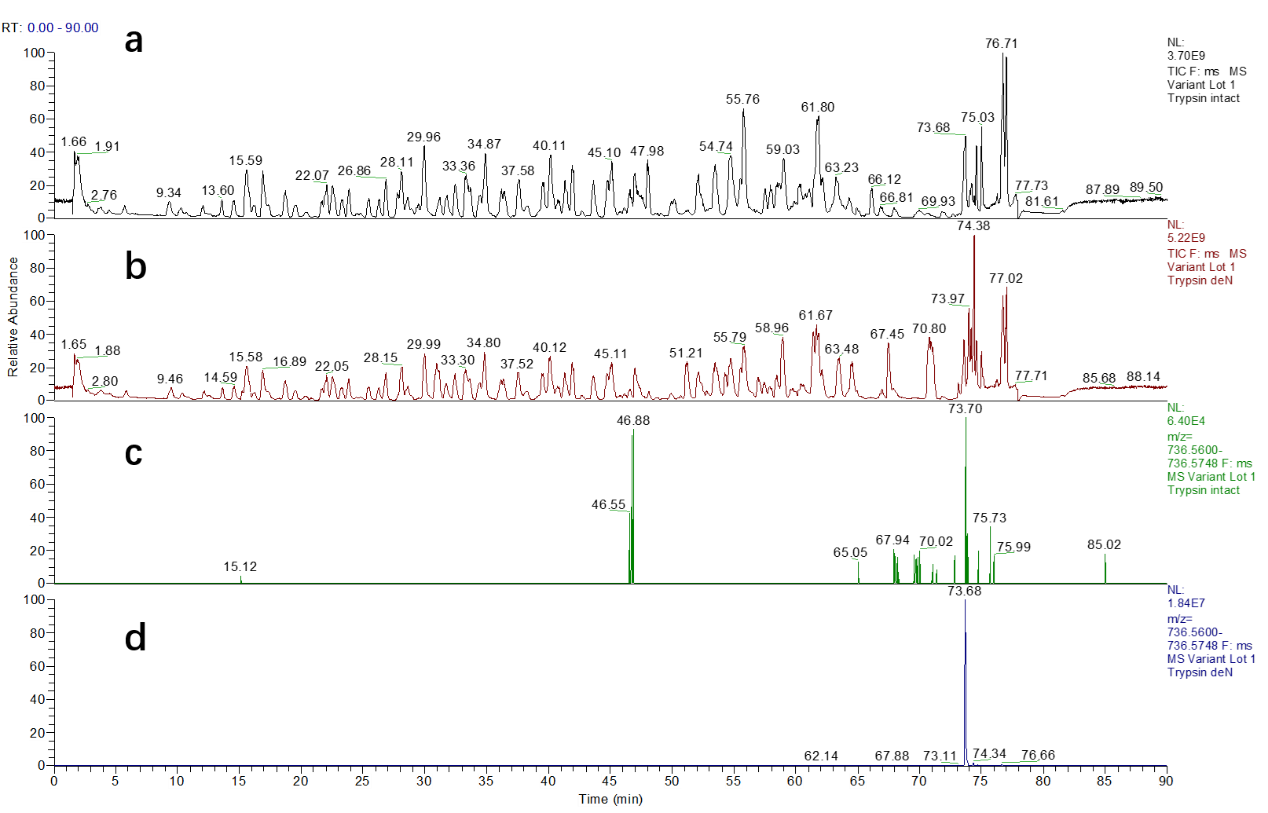


**Fig. S7** (a) Total ion chromatogram (TIC) for Lot 1 sample with tryptic digestion; (b) TIC for Lot 1 sample with PNGase F and tryptic digestion; (c) Extracted ion chromatogram (XIC) of m/z 736.5674 ± 10 ppm for Lot 1 sample with tryptic digestion; (d) XIC of m/z 736.5674 ± 10 ppm for Lot 1 sample with PNGase F and tryptic digestion.


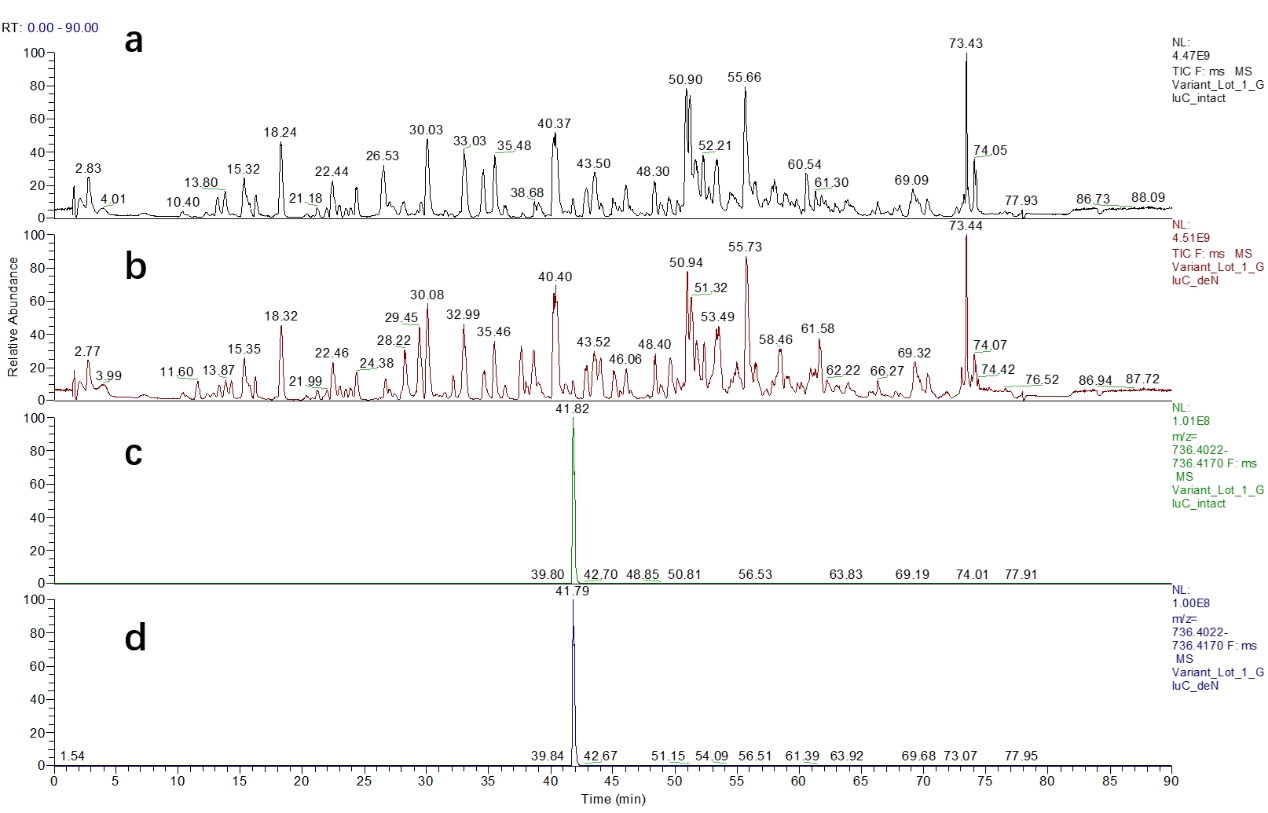


**Fig. S8** (a) Total ion chromatogram (TIC) for Lot 1 sample with GluC digestion; (b) TIC for Lot 1 sample with PNGase F and GluC digestion; (c) Extracted ion chromatogram (XIC) of m/z 736.4096 ± 10 ppm for Lot 1 sample with GluC digestion; (d) XIC of m/z 736.4096 ± 10 ppm for Lot 1 sample with PNGase F and GluC digestion.


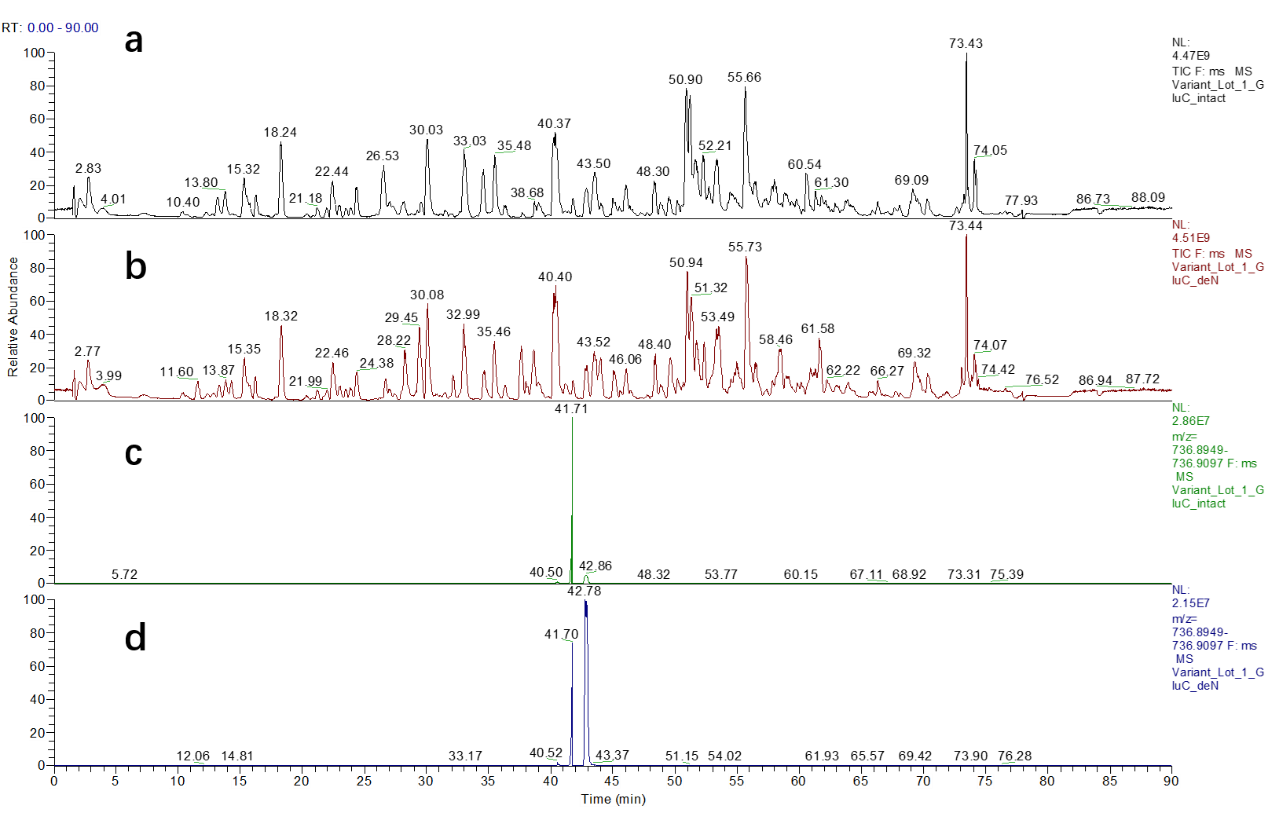


**Fig. S9** (a) Total ion chromatogram (TIC) for Lot 1 sample with GluC digestion; (b) TIC for Lot 1 sample with PNGase F and GluC digestion; (c) Extracted ion chromatogram (XIC) of m/z 736.9023 ± 10 ppm for Lot 1 sample with GluC digestion; (d) XIC of m/z 736.9023 ± 10 ppm for Lot 1 sample with PNGase F and GluC digestion.


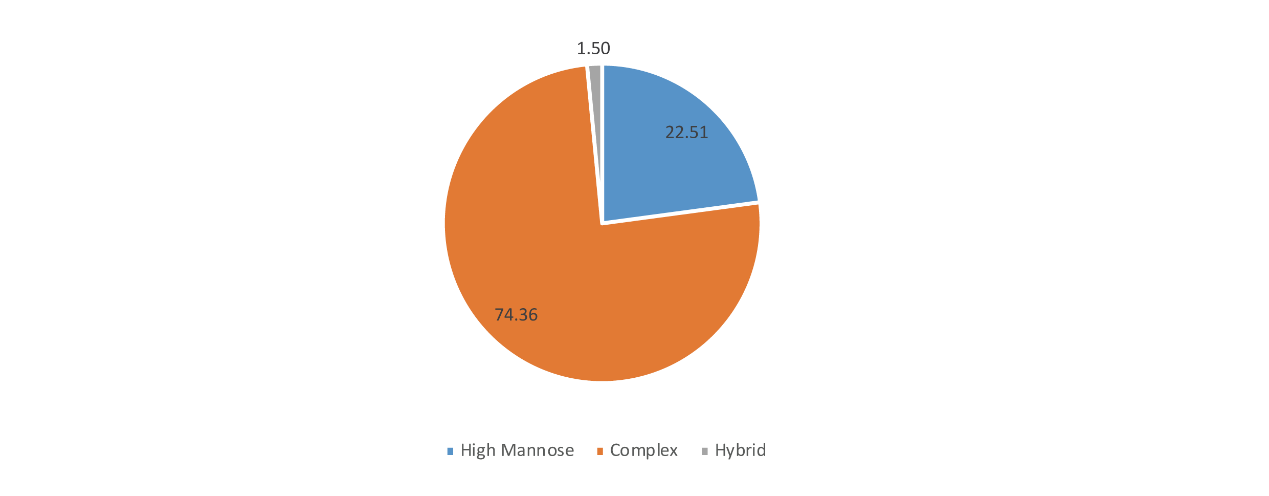


**Fig. S10** Pie chart and percentage of different N-glycan types of lot 1 N-glycan profiling data


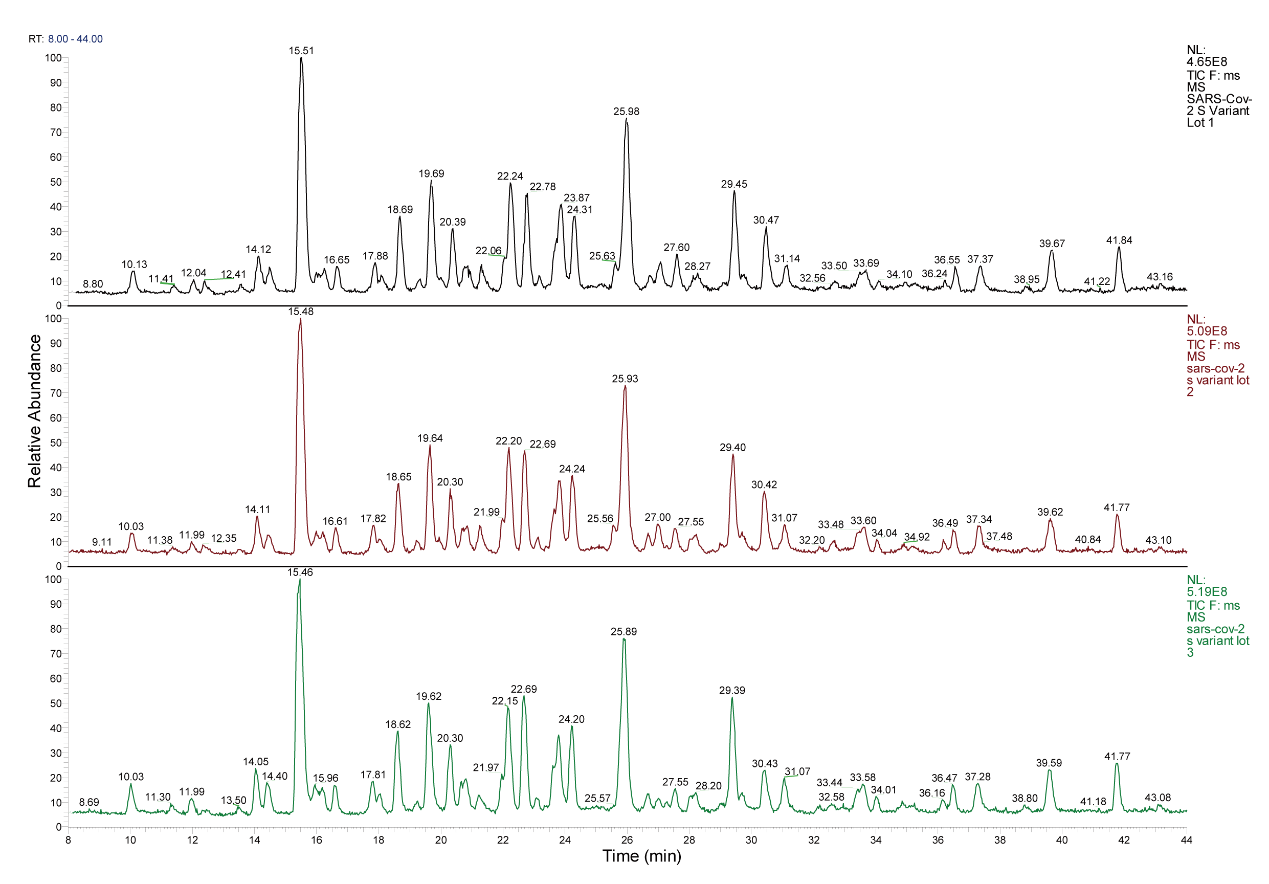


**Fig. S11** Total ion chromatograms of lot 1, lot 2 and lot 3 samples (from top to bottom) N-glycan profiling





**Fig. S12** Depiction of N-glycan percent area and variation for 3 tested Lots.
